# Internalization of Rituximab and the Efficiency of B Cell Depletion in Rheumatoid Arthritis and Systemic Lupus Erythematosus

**DOI:** 10.1002/art.39167

**Published:** 2015-07-28

**Authors:** Venkat Reddy, Geraldine Cambridge, David A. Isenberg, Martin J. Glennie, Mark S. Cragg, Maria Leandro

**Affiliations:** ^1^ University College LondonLondonUK; ^2^ Southampton UniversitySouthamptonUK

## Abstract

**Objective:**

Rituximab, a type I anti‐CD20 monoclonal antibody (mAb), induces incomplete B cell depletion in some patients with rheumatoid arthritis (RA) and systemic lupus erythematosus (SLE), thus contributing to a poor clinical response. The mechanisms of this resistance remain elusive. The purpose of this study was to determine whether type II mAb are more efficient than type I mAb at depleting B cells from RA and SLE patients, whether internalization influences the efficiency of depletion, and whether Fcγ receptor type IIb (FcγRIIb) and the B cell receptor regulate this internalization process.

**Methods:**

We used an in vitro whole blood B cell–depletion assay to assess the efficiency of depletion, flow cytometry to study cell surface protein expression, and surface fluorescence–quenching assays to assess rituximab internalization, in samples from patients with RA and patients with SLE. Paired *t*‐test or Mann‐Whitney U test was used to compare groups, and Spearman's rank correlation test was used to assess correlation.

**Results:**

We found that type II mAb internalized significantly less rituximab than type I mAb and depleted B cells from patients with RA and SLE at least 2‐fold more efficiently than type I mAb. Internalization of rituximab was highly variable between patients, was regulated by FcγRIIb, and inversely correlated with cytotoxicity in whole blood B cell–depletion assays. The lowest levels of internalization were seen in IgD– B cells, including postswitched (IgD–CD27+) memory cells. Internalization of type I anti‐CD20 mAb was also partially inhibited by anti‐IgM stimulation.

**Conclusion:**

Variability in internalization of rituximab was observed and was correlated with impaired B cell depletion. Therefore, slower‐internalizing type II mAb should be considered as alternative B cell–depleting agents for the treatment of RA and SLE.

B cell–targeted monoclonal antibodies (mAb) are increasingly being explored for use in the treatment of autoimmune diseases such as rheumatoid arthritis (RA) and systemic lupus erythematosus (SLE). Rituximab (RTX), a chimeric anti‐CD20 mAb, is licensed for the treatment of RA and is used extensively off‐label for the treatment of refractory SLE. However, RTX induces incomplete B cell depletion in some individuals with RA 1 and SLE 2, which may at least partly explain the poor clinical response noted in some individuals 3, 4. A long duration of B cell depletion in RA (using an extra dose of RTX) 5 and SLE patients is associated with better clinical response 6. Hence, enhancing B cell depletion may improve treatment efficacy, and understanding the mechanisms of resistance in RA and SLE is of clear clinical importance. B cell–depletion studies in lupus‐prone mice suggest disease‐specific mechanisms of resistance to anti‐CD20 mAb 7, but the precise mechanisms of resistance to RTX in patients with RA and SLE remain elusive.

Administration of anti‐CD20 mAb can evoke 3 main cytotoxic effector mechanisms, antibody‐dependent cell‐mediated cytotoxicity (ADCC), complement‐dependent cytotoxicity (CDC), and direct cell death 8. The association between Fcγ receptor type IIIa (FcγRIIIa) genotype and clinical response and/or the degree of B cell depletion in RA 9 and SLE 10 suggests that the ADCC‐type FcγR‐dependent systems (including antibody‐dependent cellular phagocytosis) are the main RTX effector mechanisms in vivo, in both RA and SLE, as previously noted for some B cell malignancies 11, 12, 13.

Anti‐CD20 mAb can be categorized into 2 types based on whether they redistribute CD20 into lipid rafts and consequently evoke different effector mechanisms 8, 14. Type I mAb include RTX, ofatumumab (2F2, a fully human IgG1), and ocrelizumab (a humanized IgG1). Both ofatumumab and ocrelizumab have been shown to be effective in treating patients with RA 15, 16. Type II mAb include tositumomab (anti‐B1, a mouse IgG2a) and obinutuzumab (GA101, a glycoengineered human IgG1). Type II mAb have been shown to be more efficient than type I at depleting B cells in preclinical models 17 and in patients with B cell malignancies 18, resulting in improved clinical efficacy in chronic lymphocytic leukemia 19. However, whether type II mAb are more effective at depleting B cells from patients with RA and SLE is not known.

The improved efficacy of type II mAb is attributed to the observation that normal and malignant B cells internalize type I mAb more rapidly than type II, a mechanism regulated by the inhibitory Fcγ receptor IIb (FcγRIIb) on B cells 20. Internalization of mAb reduces the ability to activate FcγR‐dependent ADCC functions 21, including phagocytosis 20, 22, and so is thought to be detrimental for target‐cell depletion, with the expression of FcγRIIb on target lymphoma cells being associated with a poor clinical response to RTX 20, 23. However, whether similar resistance mechanisms are operant in RA and SLE is not known.

In autoimmune conditions, internalization of B cell–targeted antigen–mAb‐complexes may have a beneficial immunomodulatory effect, as discussed elsewhere 24. For example, epratuzumab, an anti‐CD22 mAb, is rapidly internalized after binding to its target antigen CD22, and so anti‐CD22 mAb–conjugated toxins are used to treat B cell malignancies 25. Unconjugated anti‐CD22 mAb may have utility in autoimmune situations by facilitating endocytosis of CD22 and modulating B cell receptor (BCR) signaling 26, in addition to eliciting modest ADCC 27. Epratuzumab appears to be effective in SLE 28. B cells from patients with SLE likely internalize anti‐CD22 mAb 29; whether FcγRIIb regulates this internalization is not known.

In this study, we found that the slower‐internalizing type II anti‐CD20 mAb depleted B cells from patients with RA and SLE more efficiently than either type I anti‐CD20 or anti‐CD22 mAb and that internalization influenced the efficiency of depletion. We also found that the extent of internalization of rituximab was highly variable between patients, was regulated by FcγRIIb, and was inversely correlated with its cytotoxicity in whole blood B cell–depletion assays. Blocking of FcγRIIb inhibited the internalization of type I anti‐CD20 mAb, with variable levels of internalization noted between different B cell subpopulations; being least for postswitched (IgD–CD27+) memory cells and IgD– cells. Internalization of type I anti‐CD20, but not anti‐CD22, mAb was partially inhibited by stimulation with anti‐IgM, which suggests independent roles for the BCR and FcγRIIb in facilitating the internalization of type I mAb.

## PATIENTS AND METHODS

### Patients and healthy blood donors

Ethical approval for the study was obtained from the National Research Ethics Committee. Whole blood samples from all participants were obtained with their informed consent, adhering to the Declaration of Helsinki.

Demographic features of the RA and SLE patients are summarized in Supplementary Tables 1 and 2, respectively, available on the *Arthritis & Rheumatology* web site at http://onlinelibrary.wiley.com/doi/10.1002/art.39167/abstract. The median age of the 3 study groups was 31 years (range 22–60 years) in the healthy controls, 52 years (range 24–79 years) in the RA patients, and 39 years (range 21–76 years) in the SLE patients. All RA patients were positive for rheumatoid factor and/or anti–cyclic citrullinated peptide antibodies. Peripheral blood was collected into tubes containing lithium heparin. Peripheral blood mononuclear cells (PBMCs) were separated using Ficoll‐Paque density‐gradient centrifugation, and B cells were isolated from the PBMCs by negative selection using either a human B cell enrichment kit (StemCell Technologies) or human B cell isolation kit II (Miltenyi Biotec).

### Antibodies and reagents

AT10, which binds both FcγRIIa and FcγRIIb 30, was produced in‐house. Rituximab was a gift from the Southampton General Hospital Pharmacy, and tositumomab was a gift from Prof T. Illidge (University of Manchester, Manchester, UK). Glycosylated GA101 with an unmodified Fc portion (GA101_Gly_) and ofatumumab were produced in‐house from patented published sequences in Chinese hamster ovary or 293F cells; therefore, their carbohydrate structures may differ from mAb in clinical use. Alexa Fluor 488 and anti–Alexa Fluor 488 were purchased from Invitrogen. The mAb were labeled with Alexa Fluor 488 according to the manufacturer's (Invitrogen) instructions.

### Flow cytometry

The following fluorochrome‐conjugated mAb (all from Becton Dickinson) were used for flow cytometry: CD3 (allophycocyanin), CD19 (phycoerythrin [PE]–Cy7 or PerCP–Cy5.5), CD20 (fluorescein isothiocyanate), CD32 (PE), CD45 (PE), and IgD (Brilliant Violet 421). Flow cytometry was performed using a Becton Dickinson LSRFortessa cell analyzer. Lymphocyte populations were identified using forward‐ and side‐scatter characteristics and CD45 positivity. B cells were identified as CD19+ or CD20+ and T cells as CD3+. To account for interexperimental variation, the mean fluorescence intensity (MFI) of CD20 and FcγRIIb was determined as the ratio of the MFI of CD20/FcγRIIb to the MFI of the isotype control.

### Whole blood B cell–depletion assay

**Figure 1:**
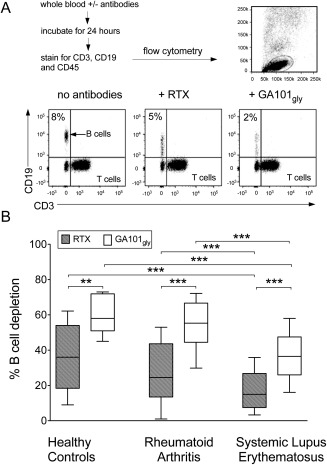
Whole blood B cell–depletion assay. **A,** Whole blood samples were incubated with or without rituximab (RTX) or glycosylated GA101 with an unmodified Fc portion (GA101_Gly_) for 24 hours, and the percentage of B cell death was determined by flow cytometry. Ten thousand events gated on lymphocytes were acquired per sample. B cells were identified as CD19+ and T cells as CD3+. **B,** Cytotoxicity (percentage B cell depletion) of RTX was significantly lower than that of GA101_Gly_ in samples from healthy controls (n = 9), patients with rheumatoid arthritis (RA; n = 26) and patients with systemic lupus erythematosus (SLE; n = 50). Cytotoxicity achieved by both RTX and GA101_Gly_ was significantly lower in SLE patients than in healthy controls or in RA patients. Data are shown as box plots. Each box represents the interquartile range. Lines inside the boxes represent the median. Whiskers represent the range. **∗∗** = *P* < 0.005; **∗∗∗** = *P* < 0.0001.

The whole blood B cell–depletion assay was performed as described previously 31. Briefly, 100 μl of freshly drawn whole blood was incubated in the presence or absence of mAb at 37°C in an atmosphere of 5% CO_2_. Samples were harvested after 24 hours and stained with anti‐CD3, anti‐CD19, and anti‐CD45 and then incubated for another 30 minutes before lysing the red blood cells with BD PharmLyse. Ten thousand events were acquired in the lymphocyte gate per sample, and the data were analyzed by flow cytometry using FlowJo software using the protocol shown in Figure 1A. The percentage of B cell depletion with mAb was defined as the cytotoxicity index (CTI) and was determined using the following formula: CTI of mAb = 100 – [(100/B cell:T cell ratio in sample without antibody) × (B cell:T cell ratio in sample with antibody)]. The percentage B cell depletion in the sample without antibody is set at 0. The mean values for triplicate wells were used to calculate the CTI.

### Surface fluorescence–quenching assay

The surface fluorescence–quenching assay was performed as described previously 22. Briefly, 2–4 × 10^5^ B cells were incubated with Alexa Fluor 488–labeled mAb in a volume of 5 μg/ml at 37°C for 6 hours. As we had observed differences in the ability of different IgG isotypes to activate FcγRIIb 20, all mAb used were either human or mouse IgG1, which give equivalent activity in internalization assays with anti‐CD20 mAb 24. Samples were then harvested, washed twice, and incubated for 30 minutes at 4°C with PE–Cy7–labeled anti‐CD19 in the presence or absence of anti–Alexa Fluor 488 quenching antibody (Invitrogen). After washing, samples were analyzed by flow cytometry.

We investigated internalization in the following B cell subpopulations: naive (IgD+CD27–), preswitched (IgD+CD27+), postswitched (IgD–CD27+), and double‐negative (IgD–CD27–) cells. Samples were stained with PE–Cy7–labeled anti‐CD19, BV421‐labeled IgD, or PE‐labeled CD27 after incubation with Alexa Fluor 488–labeled mAb.

The effect of FcγRIIb on internalization of mAb was investigated by comparing the MFI of FcγRIIb in samples with and those without prior incubation with AT10 at 50 μg/ml for 30 minutes before the addition of Alexa Fluor 488–labeled mAb. The effect of B cell activation on internalization was investigated by stimulating isolated B cells with anti‐IgM F(ab′)_2_ at 25 μg/ml for 30 minutes or for 6 hours before incubating with Alexa Fluor 488–labeled mAb.

### Statistical analysis

Statistical analyses were performed with GraphPad Prism software version 5.0. Paired‐*t* test or Mann‐Whitney U test was used to compare groups as appropriate. Spearman's rank correlation r^2^ was used to analyze correlations between parameters.

## RESULTS

### Double the efficiency of B cell depletion by type II mAb versus type I mAb

The autologous whole blood B cell–depletion assay is a comprehensive method for assessing mAb cytotoxicity in vitro, as it accounts for all 3 effector mechanisms evoked by mAb: ADCC, CDC, and direct cell death. This assay was previously used to show that type II mAb are more efficient at lysing B cells from healthy control subjects and from patients with B cell malignancies 18, 31. However, whether type II mAb are more efficient at lysing B cells from patients with autoimmune disease is not known.

Since SLE patients often have lymphopenia, making extensive assays difficult, we initially determined the optimal concentration of mAb (0.01, 0.1, 1, and 10 μg/ml) required for the assay using blood from healthy controls. In these assays, we used nonglycomodified versions of GA101 to directly assess the effects of type I versus type II mAb without the influence of afucosylation. Independent experiments were performed in whole blood samples from 4 healthy control subjects. The mean percentage of cell death was used to assess the cytotoxicity of the mAb and to determine the optimum dose. We found that GA101_Gly_ was significantly more efficient at lysing B cells than rituximab was in all 4 samples at all 4 concentrations tested (see Supplementary Figure 1, available on the *Arthritis & Rheumatology* web site at http://onlinelibrary.wiley.com/doi/10.1002/art.39167/abstract). Therefore, we used 1 μg/ml for subsequent experiments.

The cytotoxicity index from the autologous whole blood B cell–depletion assay (1 μg/ml) was calculated in whole blood samples from 9 healthy control subjects, 26 patients with RA, and 50 patients with SLE (Figure 1B). GA101_Gly_ (type II mAb) was found to be significantly more efficient than RTX (type I mAb) at lysing B cells in vitro in samples from all study groups. The mean ± SD CTI for GA101_Gly_ versus RTX was 63 ± 11 versus 36 ± 18 in healthy controls (*P* = 0.005), 54 ± 16 versus 27 ± 16 in RA patients (*P* < 0.0001), and 38 ± 15 versus 17 ± 12 in SLE patients (*P* < 0.0001). There was no significant difference between the CTI for RTX in healthy controls and RA patients, whereas the CTI for RTX was significantly lower in SLE patients as compared with healthy controls (*P* = 0.008) and RA (*P* = 0.01). Similarly, there was no significant difference in the CTI for GA101_Gly_ between healthy controls and RA patients, whereas it was significantly lower in SLE patients as compared with healthy controls (*P* = 0.0006) and with RA patients (*P* < 0.0001) (Figure 1B). The median ratio of the CTI for GA101_Gly_ to the CTI for RTX was 1.5, 1.7, and 2.5, for healthy controls, RA patients, and SLE patients, respectively.

We did not find a correlation between the CTI for RTX and the distribution of relative frequencies of B cell subpopulations (data not shown and Supplementary Table 3, available on the *Arthritis & Rheumatology* web site at http://onlinelibrary.wiley.com/doi/10.1002/art.39167/abstract.) or serum complement C3 levels (data not shown). Taken together, these results suggested that type II mAb are more effective at depleting B cells in each subset of study subjects and that B cells from SLE patients are less susceptible to lysis by RTX and by GA101_Gly_, indicating an inherent resistance mechanism.

**Figure 2:**
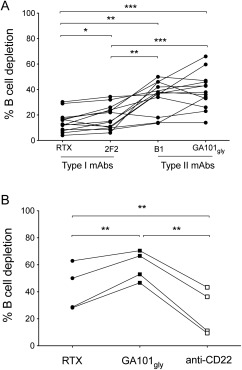
Efficiency of B cell lysis by type I monoclonal antibody (mAb) as compared with type II mAb and with anti‐CD22 mAb. **A,** Whole blood samples were incubated with or without 1 μg/ml of either rituximab (RTX; IgG1), ofatumumab (2F2; IgG1), tositumomab (B1; mouse IgG2a), or glycosylated GA101 with an unmodified Fc portion (GA101_Gly_; IgG1). After 24 hours, the percentage of B cell death was measured by flow cytometry. The cytotoxicity of type I and type II mAb was compared in patients with rheumatoid arthritis (n = 3) and systemic lupus erythematosus (SLE; n = 10). Type I mAb lysed B cells less efficiently than did type II mAb, with a cytotoxicity index for RTX < 2F2 < B1 < GA101_Gly_. Values are the mean of triplicate wells. Each line represents an individual sample. **B,** In SLE patients (n = 4), the cytotoxicity index of anti‐CD22mAb was significantly lower than that of RTX and GA101_Gly_. **∗** = *P* < 0.05; **∗∗** = *P* < 0.005; **∗∗∗** = *P* < 0.0001.

Next, we wanted to investigate whether the difference in the B cell–lysing potential of RTX and GA101_Gly_ was also applicable to additional type I and type II mAb. We therefore compared the CTI of 2 other mAb: ofatumumab (2F2) and tositumomab (B1), representing type I and type II mAb, respectively (Figure 2A). Again, we found that type II mAb were significantly more efficient than type I mAb at lysing B cells in all samples examined from patients with RA (n = 3) and SLE (n = 10). We noted a significant hierarchy in the efficiency of CTI of the mAb, with GA101_Gly_ > B1 >2F2 > RTX and with a >2‐fold difference in the CTIs for GA101_Gly_ versus 2F2 (Figure 2A).

As anti‐CD22 mAb have also been reported to deplete B cells, albeit weakly 27, we examined their activity in the assay. We found that the CTI for anti‐CD22 mAb was found to be significantly lower than that for anti‐CD20 mAb, with a CTI hierarchy of anti‐CD22 < RTX < GA101_Gly_ (n = 4) (Figure 2B). This may be at least partly due to the differences in internalization of the mAb, as noted previously for B cell malignancies 24.

Both B cell–intrinsic and B cell–extrinsic factors may account for the apparent resistance of SLE B cells to depletion. Malignant B cell expression of CD20 32 and FcγRIIb 20 correlated with susceptibility to deletion by anti‐CD20 mAb; however, we did not find a correlation between the expression of CD20 and FcγRIIb or between their relative expression (ratio of the MFI of CD20 to the MFI of FcγRIIb) and the CTI for RTX or GA101_Gly_ in all groups examined (data not shown). This may reflect the relatively small difference in B cell expression of CD20 and FcγRIIb between these RA and SLE study patients (data not shown), in contrast to that reported for patients with B cell malignancies 20, 32.

### Efficiency of depletion influenced by internalization of rituximab

**Figure 3:**
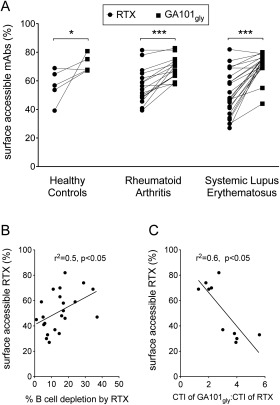
Internalization of rituximab (RTX) to a highly variable extent, impairing its efficiency of depletion. **A,** Internalization was assessed by surface fluorescence–quenching assay, which revealed that a greater percentage of glycosylated GA101 with an unmodified Fc portion (GA101_Gly_) than RTX was accessible for quenching in samples from healthy controls (n = 5), rheumatoid arthritis patients (n = 16), and systemic lupus erythematosus (SLE) patients (n = 22). Each line represents an individual sample. **∗** = *P* < 0.05; **∗∗∗** = *P* < 0.0001. **B,** Spearman's rank correlation analysis showed a significant correlation between the percentage of surface‐accessible RTX and the percentage of B cell depletion in patients with SLE (n = 22), as assessed by whole blood B cell–depletion assay. **C,** The relative cytotoxicity and the ratio of the cytotoxicity index (CTI) for GA101_Gly_ to the CTI for RTX between SLE patients with >65% surface‐accessible RTX and SLE patients with <40% surface‐accessible RTX was 2‐fold and 4‐fold, respectively, and correlated with the percentage of surface‐accessible RTX, by Spearman's rank correlation analysis. mAbs = monoclonal andibodies.

Given the large variability in depletion afforded by RTX in SLE and a superior efficacy of type II mAb in the whole blood B cell–depletion assays, we next examined whether internalization of mAb might explain the greater resistance of SLE B cells to depletion. Internalization was assessed using the surface fluorescence–quenching assay using isolated B cells from 5 healthy controls, 16 patients with RA, and 22 patients with SLE. In all groups, a significantly greater percentage of GA101_Gly_ than RTX was accessible on the cell surface. The median percentage of surface‐accessible mAb after 6 hours of incubation for GA101_Gly_ versus RTX in the healthy controls, RA patients, and SLE patients was 67 versus 57, 69 versus 55 (*P* < 0.005), and 74 versus 47 (*P* < 0.005), respectively (Figure 3A). Thus, internalization of mAb was a notable feature of B cells from healthy controls as well as from patients with RA and SLE.

Interestingly, we noted a correlation between surface‐accessible RTX and the CTI for RTX (Spearman's r^2^ = 0.5, *P* < 0.05) (Figure 3B) and between the relative CTI for GA101_Gly_ and the relative CTI for RTX (Spearman's r^2^ = 0.6, *P* < 0.05) in samples from SLE patients, but not those from healthy controls or RA patients (data not shown).

The relative potency of RTX compared with GA101_Gly_ also differed, such that in samples with >65% surface‐accessible RTX (n = 5), the mean difference in relative potency was 2‐fold, whereas in samples with <40% surface‐accessible RTX (n = 5), the mean difference in relative potency was 4‐fold (Figure 3C). We found no significant correlations between surface‐accessible GA101_Gly_ and the CTI for GA101_Gly_ in all groups examined (data not shown). The results therefore suggest that internalization of RTX contributes to its inferior efficiency of depletion, as assessed by whole blood B cell–depletion assay, in B cells from SLE patients.

### FcγRIIb facilitation of rituximab internalization

**Figure 4:**
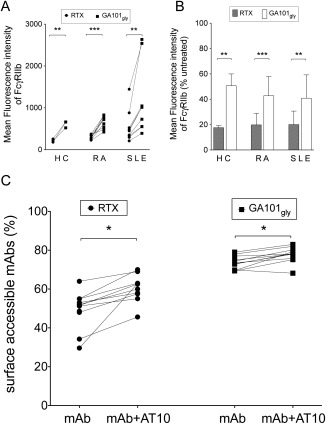
Fcγ receptor type IIb (FcγRIIb) regulation of the internalization of rituximab (RTX). **A,** The mean fluorescence intensity (MFI) of FcγRIIb was significantly lower in samples incubated with RTX than in those incubated with glycosylated GA101 with an unmodified Fc portion (GA101_Gly_) in all samples from healthy control (HC) subjects (n = 3), rheumatoid arthritis (RA) patients (n = 9), and systemic lupus erythematosus (SLE) patients (n = 9), suggesting that RTX was internalized along with FcγRIIb. Each line represents an individual sample. **B,** The MFI of FcγRIIb in samples incubated with monoclonal antibodies (mAb) compared with that in samples without antibodies, expressed as a percentage of untreated samples, revealed significantly lower values in samples incubated with RTX as compared with those incubated with GA101_Gly_ in all 3 study groups. Values are the mean ± SD. **C,** Blocking of FcγRIIb using AT10 (an anti‐FcγRII mAb) inhibited the internalization of RTX to a greater extent than that of GA101_Gly_ in SLE patients (n = 11). Each line represents an individual sample. **∗** = *P* < 0.05; **∗∗** = *P* < 0.005; **∗∗∗** = *P* < 0.0001.

RTX internalizes as part of a tripartite complex with CD20 and FcγRIIb 20, but B cell expression of FcγRIIb may be altered in SLE 33. We therefore investigated whether FcγRIIb also regulated the internalization of mAb in samples from RA and SLE patients and whether FcγRIIb internalized to a greater extent with RTX than with GA101_Gly_. Isolated B cells from 3 healthy controls, 9 RA patients, and 9 SLE patients were incubated for 6 hours in the presence or absence of 5 μg/ml of mAb. A significant difference in the mean fluorescence intensity of FcγRIIb (*P* < 0.005 for each comparison) was seen in all 3 groups (Figures 4A and B), with RTX having the greatest internalization.

We then examined whether blocking FcγRIIb inhibited internalization in samples from 11 patients with SLE, with or without prior incubation with AT10 (an FcγRII‐specific mAb) 30. Internalization of both RTX and GA101_Gly_ was inhibited by AT10. However, accessible RTX was greater in samples incubated with AT10 as compared with samples without AT10 (median 61% versus 51%, respectively) whereas this difference was only modest for GA101_Gly_ (median 78% versus 74%) (Figure 4C). Intriguingly, despite blocking FcγRIIb, the median surface‐accessible RTX was lower than that of GA101_Gly_ (61% versus 78%). Although there was no direct correlation between the degree of inhibition of internalization with AT10 or between the MFI of FcγRIIb and the fold difference between the CTI for RTX and the CTI for GA101_Gly_, we noted that in the 2 samples with the greatest inhibition of mAb internalization, the CTI for GA101_Gly_ was >4‐fold higher than that for RTX, whereas the mean for the cohort was a 2‐fold difference in CTI between the 2 mAb (data not shown). Thus, FcγRIIb facilitated the internalization of type I CD20 mAb and reduced the efficiency of deletion, albeit to a variable extent.

### Disparity in internalization of B cell–targeted mAb

**Figure 5:**
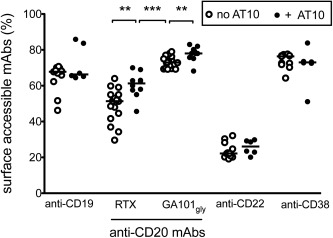
Disparity in the internalization of monoclonal antibodies (mAb) and inhibition by Fcγ receptor type IIb (FcγRIIb). There was a high rate of internalization of anti‐CD22 mAb, as assessed by the surface fluorescence–quenching assay. Internalization of rituximab (RTX; a type I anti‐CD20 mAb) and, to some extent, anti‐CD19 mAb, showed remarkable variability between samples, whereas internalization of anti‐CD38 mAb and glycosylated GA101 with an unmodified Fc portion (GA101_Gly_) was consistently low. Internalization of only type I and type II anti‐CD20 mAb, but not the other mAb, was significantly inhibited by anti‐FcγRII mAb (AT10). Each symbol represents an individual sample; horizontal lines show the median. **∗∗** = *P* < 0.005; **∗∗∗** = *P* < 0.0001.

In addition to CD20, mAb targeting other B cell surface antigens are being explored for use in SLE, including CD19 34 and CD22 28. We therefore investigated whether the differences in the CTIs for type I and type II CD20 mAb and anti‐CD22 mAb (Figure 2B) were due to a disparity in internalization and whether FcγRIIb regulated their internalization. The median percentages of surface‐accessible mAb were 67%, 51%, 73%, 22%, and 76% for anti‐CD19, type I anti‐CD20 (RTX), type II anti‐CD20 (GA101_Gly_), anti‐CD22, and anti‐CD38 mAb, respectively (Figure 5). Furthermore, similar to our observations in malignant B cells 24, SLE B cells also displayed a remarkable degree of internalization of anti‐CD22 mAb, greater than that seen with RTX, whereas the other mAb (anti‐CD19, anti‐CD38, and GA101_Gly_) were internalized to a lesser degree. In contrast to the hierarchy of depletion with mAb, with anti‐CD22 < RTX < GA101_Gly_ (Figure 2B), we noted a reverse hierarchy of the extent of internalization, with anti‐CD22 > RTX > GA101_Gly_. However, only internalization of anti‐CD20 mAb was consistently inhibited by AT10 and was therefore FcγRIIb‐dependent.

### Influence of IgD and B cell activation on the internalization of type I mAb

**Figure 6:**
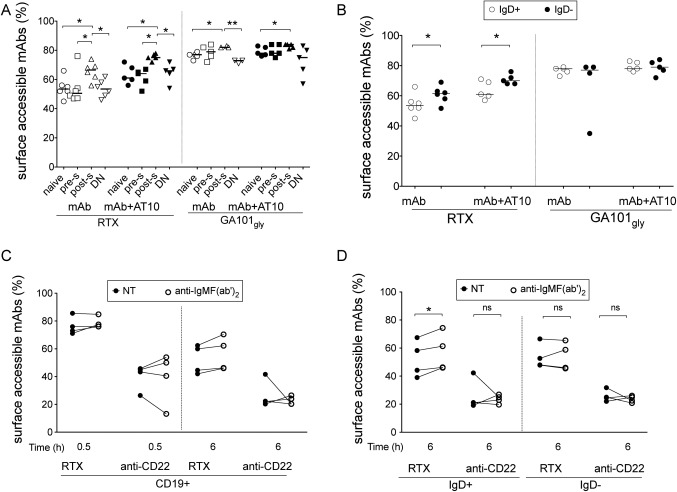
Effect of IgD and B cell activation on the internalization of anti‐CD20 monoclonal antibodies (mAb) in B cell subpopulations. **A,** Rituximab (RTX) was internalized to a significantly lesser extent by postswitched (post‐s) memory cells (IgD–CD27+) than by the other B cell subpopulations, both before and after blocking with AT10. A significantly greater percentage of glycosylated GA101 with an unmodified Fc portion (GA101_Gly_) was accessible on postswitched cells than on naive (IgD+CD27–) or double‐negative (DN; IgD–CD27–) cells before blocking with AT10 and only on naive cells after blocking with AT10. Preswitched (pre‐s) cells were defined as IgD+CD27+ B cells. **B,** A greater percentage of RTX was accessible on the surface of IgD– B cells than on IgD+ B cells. No such difference was noted for GA101_Gly_. **C,** Internalization of RTX was not inhibited by B cell activation with anti–IgM F(ab′)_2_ in CD19+ B cells as compared with no treatment (NT). **D,** A greater percentage of RTX was accessible at 6 hours in IgD+ B cells, but not IgD– B cells, from samples incubated with anti‐IgM F(ab′)_2_ as compared with untreated samples. No such difference was noted for anti‐CD22 mAb. In **A** and **B,** each symbol represents an individual sample; horizontal lines show the median. In **C** and **D,** each line represents an individual sample. **∗** = *P* < 0.05; **∗∗** = *P* < 0.005. NS = not significant.

We next examined whether there were any differences in internalization between B cell subpopulations in samples from 5 patients with SLE. In all cases, postswitched memory cells internalized significantly less RTX than did naive, preswitched, and double‐negative cells (*P* < 0.05 for each comparison) (Figure 6A). For GA101_Gly_, a significant difference was noted between postswitched cells and naive and double‐negative cells before blocking with AT10 and only in naive cells after blocking with AT10.

We also examined differences in internalization between B cell subpopulations based on the expression of IgD (IgD+ or IgD–), CD27 (CD27+ or CD27–), and CD38 (CD38^low^ or CD38++). Again, in samples incubated with RTX, there was significantly greater internalization of RTX in IgD+ B cells than IgD– B cells, and internalization was inhibited by AT10 (Figure 6B). This may be partly due to the differential expression of FcγRIIb and IgD (Supplementary Figures 2A and B, available on the *Arthritis & Rheumatology* web site at http://onlinelibrary.wiley.com/doi/10.1002/art.39167/abstract). No such findings were observed in B cell subpopulations based on the expression of CD27 (Supplementary Figure 3A, available on the *Arthritis & Rheumatology* web site at http://onlinelibrary.wiley.com/doi/10.1002/art.39167/abstract) or CD38 (Supplementary Figure 3B). In samples incubated with GA101_Gly_, no differences were observed with any B cell subpopulations. We found no differences in the internalization of anti‐CD22 mAb between B cell subpopulations (n = 3) (data not shown). Thus, internalization of type I mAb, but not type II mAb or anti‐CD22 mAb, was significantly lower in postswitched cells and IgD– B cells overall.

It has previously been reported that the expression of FcγRIIb differs between B cell subpopulations in SLE patients 33 and may therefore account for the disparity in internalization. We confirmed that the expression of FcγRIIb varied between B cell subpopulations in SLE (Supplementary Figure 2A, available on the *Arthritis & Rheumatology* web site at http://onlinelibrary.wiley.com/doi/10.1002/art.39167/abstract), with naive cells < double‐negative cells < postswitched cells < preswitched cells. In contrast to the expression of FcγRIIb, the expression of IgD on naive cells was greater than that in preswitched cells (Supplementary Figure 2B). This finding was especially of interest in conjunction with the finding that internalization of mAb was greatest in the IgD+ B cells rather than the IgD– B cells.

Given that internalization of mAb was higher in the IgD+ B cells and that the outcome of BCR engagement leading to either signaling or internalization has previously been shown to be mutually exclusive and dependent on the phosphorylation of tyrosine‐based motifs 35, we investigated whether B cell activation inhibited internalization of RTX or anti‐CD22 mAb. Isolated B cells were incubated with or without 25 μg/ml of anti‐IgM F(ab′)_2_ for 0.5 or 6 hours. Internalization of RTX, but not anti‐CD22 mAb, was inhibited by B cell activation with anti‐IgM F(ab′)_2_ only in IgD+ B cells (*P* < 0.05), but not IgD− B cells, at 6 hours (Figures 6C and D). Taken together, these results suggest independent roles for FcγRIIb and the BCR in regulating the internalization of RTX, but not GA101_Gly_ or anti‐CD22 mAb.

## DISCUSSION

Rituximab treatment was first used at our center for the treatment of RA 36 and SLE 37. Although efficacy was demonstrated in seropositive RA 38 and despite encouraging results in several open studies 39, 2 randomized clinical trials failed to show efficacy in SLE 40, 41. We have previously discussed whether several factors, including trial design, may have contributed to the apparent lack of efficacy in these trials 42. A key factor is that rituximab fails to induce complete depletion in some patients with RA 3, 43 and SLE 4, which is associated with a poor treatment response. We have previously shown that serum rituximab levels vary remarkably in both RA and SLE patients, are higher in RA patients than in SLE patients, and are higher in patients with well‐depleted B cells than in those without, but only in RA patients and not SLE patients 44.

Together, these findings suggested disease‐specific mechanisms of resistance to depletion, in particular for SLE, and enhancing depletion may improve clinical response. Furthermore, a better understanding of resistance mechanisms may guide selection of appropriate B cell–depleting agents. Our goal in the present study was thus to compare the in vitro efficiency of RTX and alternative CD20 mAb and to explore potential resistance mechanisms in RA and SLE.

We used whole blood B cell–depletion assays to compare the type I mAb RTX and ofatumumab with the type II mAb tositumomab (B1) and GA101_Gly_ and showed that the type II mAb were significantly more effective at depleting B cells from patients with RA and SLE. Owing to its murine IgG2a isotype, tositumomab would be expected to be less efficient at recruiting CDC and ADCC in humans as compared with the human IgG1 isotype of rituximab; however, the type II nature appears to offset the murine isotype effect, resulting in superior cytotoxicity to that of RTX in the whole blood B cell–depletion assay.

A wide variability in the efficiency of depletion was observed in the case of RTX in SLE patients, which correlated with the level of internalization. This suggested that internalization of RTX is a probable “resistance mechanism” in patients with SLE and may explain its variability in depletion 45. The activity of the 2 types of mAb demonstrated in vitro may not reflect their activity in vivo. However, alterations of the immune system in patients with SLE, such as defective phagocytosis 46 and natural killer cell function 47, may explain why even type II anti‐CD20 mAb failed to achieve B cell depletion in SLE patients that was comparable to that in RA patients and healthy controls. Furthermore, type I CD20 mAb (RTX) induces CDC, whereas type II mAb are poor inducers of CDC 8, and thus, the efficiency of type I CD20 mAb may be compromised in conditions with defects in complement function, such as SLE 48. We are currently investigating this possibility.

The development of human antichimeric antibodies is more common in SLE patients 2, and this limits the repeated use of RTX. GA101, a fully humanized, Fc‐engineered type II CD20 mAb that has been shown to achieve better patient outcomes than RTX in chronic lymphocytic leukemia 19, which is now a Food and Drug Administration–approved indication 49, may potentially overcome, at least in part, these resistance mechanisms.

Also under exploration for use in SLE are mAb that target B cell surface proteins other than CD20, including anti‐CD19 34 and anti‐CD22 mAb 50, which are aimed at depleting B cells and/or modulating their function. We found a differential internalization of these mAb in B cells from patients with SLE, with rapid internalization of anti‐CD22 mAb that was unaffected by FcγRIIb and with variable internalization and regulation of anti‐CD19 mAb by FcγRIIb. Internalization of mAb results in lower amounts of mAb on the target cell surface being accessible to immune effector cells 22, thereby compromising their cytotoxicity, particularly in SLE, which showed rapid internalization of RTX.

Interestingly, internalization of RTX, but not anti‐CD22 mAb, was variable across B cell subpopulations, being low in postswitched (IgD–CD27+) memory cells and IgD– cells, which suggests reduced intrinsic resistance to depletion. Also, internalization of RTX, but not anti‐CD22 mAb, was independently inhibited both by blocking of FcγRIIb and by B cell activation in IgD+ B cells. Taken together, these results suggest that FcγRIIb and BCR activation influence the internalization of type I anti‐CD20 mAb, but not anti‐CD22 mAb. Thus, distinct mechanisms operate to facilitate the internalization of different mAb. The differences in internalization between antigen‐specific mAb may be related to the constitutive endocytosis of the target antigen, as for CD22 51, or to the redistribution of CD20 into lipid rafts after incubation with RTX 22. Knowledge of the factors that influence internalization of mAb could be exploited to refine B cell–targeting strategies in autoimmune diseases such as RA and SLE.

In conclusion, our results provide strong preclinical evidence for considering the use of mechanistically different type II CD20 mAb such as GA101 as alternative B cell–depleting agents for the treatment of RA and SLE. We have also identified distinct mechanisms of internalization of rituximab and its regulation, which may explain the variability in B cell depletion noted in patients with SLE.

## AUTHOR CONTRIBUTIONS

All authors were involved in drafting the article or revising it critically for important intellectual content, and all authors approved the final version to be published. Dr. Reddy had full access to all of the data in the study and takes responsibility for the integrity of the data and the accuracy of the data analysis.

### Study conception and design

Reddy, Isenberg, Glennie, Cragg, Leandro.

### Acquisition of data

Reddy.

### Analysis and interpretation of data

Reddy, Cambridge, Isenberg, Cragg, Leandro.

## Supporting information

Supplementary Figure 1. Dose‐response experiments. We determined the optimal concentration of mAbs (0.01, 0.1, 1 and 10 μg/mL) in four Independent experiments using blood from normal healthy controls and using non‐glycomodified versions of GA101 (GA101_gly_) to directly assess the effects of type I versus II without the influence of afucosylation. Whole blood samples were incubated with or without RTX or GA101_gly_ at 0.01, 0.1, 1 and 10 μg/ml and percentage B cell death measured by flow cytometric analysis after 24 h and mean of triplicate wells was used. Cytotoxicity of RTX and GA101_gly_ were compared in healthy controls (n=4). Rituximab (RTX) lyses B cells less efficiently than GA101_gly_ in all four samples at all four concentrations tested. The results are the means and SD.
Supplementary Figure 2. Differential expression of IgD and FcγRIIb in B cell subpopulations. (A) Similar to a previous report,PEVuZE5vdGU+PENpdGU+PEF1dGhvcj5NYWNrYXk8L0F1dGhvcj48WWVhcj4yMDA2PC9ZZWFyPjxSZWNOdW0+MTk1NDg8L1JlY051bT48RGlzcGxheVRleHQ+PHN0eWxlIGZhY2U9InN1cGVyc2NyaXB0Ij4xPC9zdHlsZT48L0Rpc3BsYXlUZXh0PjxyZWNvcmQ+PHJlYy1udW1iZXI+MTk1NDg8L3JlYy1udW1iZXI+PGZvcmVpZ24ta2V5cz48a2V5IGFwcD0iRU4iIGRiLWlkPSJ6eHNhejV0YWJ4ZGY1N2U1cDJocHp4NXVwMnBzZWFkNWFyc2EiPjE5NTQ4PC9rZXk+PC9mb3JlaWduLWtleXM+PHJlZi10eXBlIG5hbWU9IkpvdXJuYWwgQXJ0aWNsZSI+MTc8L3JlZi10eXBlPjxjb250cmlidXRvcnM+PGF1dGhvcnM+PGF1dGhvcj5NYWNrYXksIE0uPC9hdXRob3I+PGF1dGhvcj5TdGFuZXZza3ksIEEuPC9hdXRob3I+PGF1dGhvcj5XYW5nLCBULjwvYXV0aG9yPjxhdXRob3I+QXJhbm93LCBDLjwvYXV0aG9yPjxhdXRob3I+TGksIE0uPC9hdXRob3I+PGF1dGhvcj5Lb2VuaWcsIFMuPC9hdXRob3I+PGF1dGhvcj5SYXZldGNoLCBKLiBWLjwvYXV0aG9yPjxhdXRob3I+RGlhbW9uZCwgQi48L2F1dGhvcj48L2F1dGhvcnM+PC9jb250cmlidXRvcnM+PGF1dGgtYWRkcmVzcz5EZXBhcnRtZW50IG9mIE1lZGljaW5lLCBDb2x1bWJpYSBVbml2ZXJzaXR5IE1lZGljYWwgQ2VudGVyLCBOZXcgWW9yaywgTlkgMTAwMzIsIFVTQS4gbWNtMjEyM0Bjb2x1bWJpYS5lZHU8L2F1dGgtYWRkcmVzcz48dGl0bGVzPjx0aXRsZT5TZWxlY3RpdmUgZHlzcmVndWxhdGlvbiBvZiB0aGUgRmNnYW1tYUlJQiByZWNlcHRvciBvbiBtZW1vcnkgQiBjZWxscyBpbiBTTEU8L3RpdGxlPjxzZWNvbmRhcnktdGl0bGU+SiBFeHAgTWVkPC9zZWNvbmRhcnktdGl0bGU+PGFsdC10aXRsZT5UaGUgSm91cm5hbCBvZiBleHBlcmltZW50YWwgbWVkaWNpbmU8L2FsdC10aXRsZT48L3RpdGxlcz48cGVyaW9kaWNhbD48ZnVsbC10aXRsZT5KIEV4cCBNZWQ8L2Z1bGwtdGl0bGU+PGFiYnItMT5UaGUgSm91cm5hbCBvZiBleHBlcmltZW50YWwgbWVkaWNpbmU8L2FiYnItMT48L3BlcmlvZGljYWw+PGFsdC1wZXJpb2RpY2FsPjxmdWxsLXRpdGxlPkogRXhwIE1lZDwvZnVsbC10aXRsZT48YWJici0xPlRoZSBKb3VybmFsIG9mIGV4cGVyaW1lbnRhbCBtZWRpY2luZTwvYWJici0xPjwvYWx0LXBlcmlvZGljYWw+PHBhZ2VzPjIxNTctNjQ8L3BhZ2VzPjx2b2x1bWU+MjAzPC92b2x1bWU+PG51bWJlcj45PC9udW1iZXI+PGVkaXRpb24+MjAwNi8wOC8yMzwvZWRpdGlvbj48a2V5d29yZHM+PGtleXdvcmQ+QWR1bHQ8L2tleXdvcmQ+PGtleXdvcmQ+QWZyaWNhbiBBbWVyaWNhbnM8L2tleXdvcmQ+PGtleXdvcmQ+QW5pbWFsczwva2V5d29yZD48a2V5d29yZD5CLUx5bXBob2N5dGUgU3Vic2V0cy9jeXRvbG9neS8qaW1tdW5vbG9neS9waHlzaW9sb2d5PC9rZXl3b3JkPjxrZXl3b3JkPkNhbGNpdW0vbWV0YWJvbGlzbTwva2V5d29yZD48a2V5d29yZD5GZW1hbGU8L2tleXdvcmQ+PGtleXdvcmQ+SHVtYW5zPC9rZXl3b3JkPjxrZXl3b3JkPipJbW11bm9sb2dpYyBNZW1vcnk8L2tleXdvcmQ+PGtleXdvcmQ+THVwdXMgRXJ5dGhlbWF0b3N1cywgU3lzdGVtaWMvKmltbXVub2xvZ3k8L2tleXdvcmQ+PGtleXdvcmQ+THltcGhvY3l0ZSBBY3RpdmF0aW9uPC9rZXl3b3JkPjxrZXl3b3JkPk1hbGU8L2tleXdvcmQ+PGtleXdvcmQ+TWljZTwva2V5d29yZD48a2V5d29yZD5NaWRkbGUgQWdlZDwva2V5d29yZD48a2V5d29yZD5SZWNlcHRvcnMsIEFudGlnZW4sIEItQ2VsbC9pbW11bm9sb2d5PC9rZXl3b3JkPjxrZXl3b3JkPlJlY2VwdG9ycywgSWdHLyptZXRhYm9saXNtPC9rZXl3b3JkPjwva2V5d29yZHM+PGRhdGVzPjx5ZWFyPjIwMDY8L3llYXI+PHB1Yi1kYXRlcz48ZGF0ZT5TZXAgNDwvZGF0ZT48L3B1Yi1kYXRlcz48L2RhdGVzPjxpc2JuPjAwMjItMTAwNyAoUHJpbnQpJiN4RDswMDIyLTEwMDcgKExpbmtpbmcpPC9pc2JuPjxhY2Nlc3Npb24tbnVtPjE2OTIzODQ5PC9hY2Nlc3Npb24tbnVtPjx3b3JrLXR5cGU+UmVzZWFyY2ggU3VwcG9ydCwgTi5JLkguLCBFeHRyYW11cmFsJiN4RDtSZXNlYXJjaCBTdXBwb3J0LCBOb24tVS5TLiBHb3YmYXBvczt0PC93b3JrLXR5cGU+PHVybHM+PHJlbGF0ZWQtdXJscz48dXJsPmh0dHA6Ly93d3cubmNiaS5ubG0ubmloLmdvdi9wdWJtZWQvMTY5MjM4NDk8L3VybD48dXJsPmh0dHA6Ly9qZW0ucnVwcmVzcy5vcmcvY29udGVudC8yMDMvOS8yMTU3LmZ1bGwucGRmPC91cmw+PC9yZWxhdGVkLXVybHM+PC91cmxzPjxjdXN0b20yPjIxMTgzOTA8L2N1c3RvbTI+PGVsZWN0cm9uaWMtcmVzb3VyY2UtbnVtPjEwLjEwODQvamVtLjIwMDUxNTAzPC9lbGVjdHJvbmljLXJlc291cmNlLW51bT48bGFuZ3VhZ2U+ZW5nPC9sYW5ndWFnZT48L3JlY29yZD48L0NpdGU+PC9FbmROb3RlPn==PEVuZE5vdGU+PENpdGU+PEF1dGhvcj5NYWNrYXk8L0F1dGhvcj48WWVhcj4yMDA2PC9ZZWFyPjxSZWNOdW0+MTk1NDg8L1JlY051bT48RGlzcGxheVRleHQ+PHN0eWxlIGZhY2U9InN1cGVyc2NyaXB0Ij4xPC9zdHlsZT48L0Rpc3BsYXlUZXh0PjxyZWNvcmQ+PHJlYy1udW1iZXI+MTk1NDg8L3JlYy1udW1iZXI+PGZvcmVpZ24ta2V5cz48a2V5IGFwcD0iRU4iIGRiLWlkPSJ6eHNhejV0YWJ4ZGY1N2U1cDJocHp4NXVwMnBzZWFkNWFyc2EiPjE5NTQ4PC9rZXk+PC9mb3JlaWduLWtleXM+PHJlZi10eXBlIG5hbWU9IkpvdXJuYWwgQXJ0aWNsZSI+MTc8L3JlZi10eXBlPjxjb250cmlidXRvcnM+PGF1dGhvcnM+PGF1dGhvcj5NYWNrYXksIE0uPC9hdXRob3I+PGF1dGhvcj5TdGFuZXZza3ksIEEuPC9hdXRob3I+PGF1dGhvcj5XYW5nLCBULjwvYXV0aG9yPjxhdXRob3I+QXJhbm93LCBDLjwvYXV0aG9yPjxhdXRob3I+TGksIE0uPC9hdXRob3I+PGF1dGhvcj5Lb2VuaWcsIFMuPC9hdXRob3I+PGF1dGhvcj5SYXZldGNoLCBKLiBWLjwvYXV0aG9yPjxhdXRob3I+RGlhbW9uZCwgQi48L2F1dGhvcj48L2F1dGhvcnM+PC9jb250cmlidXRvcnM+PGF1dGgtYWRkcmVzcz5EZXBhcnRtZW50IG9mIE1lZGljaW5lLCBDb2x1bWJpYSBVbml2ZXJzaXR5IE1lZGljYWwgQ2VudGVyLCBOZXcgWW9yaywgTlkgMTAwMzIsIFVTQS4gbWNtMjEyM0Bjb2x1bWJpYS5lZHU8L2F1dGgtYWRkcmVzcz48dGl0bGVzPjx0aXRsZT5TZWxlY3RpdmUgZHlzcmVndWxhdGlvbiBvZiB0aGUgRmNnYW1tYUlJQiByZWNlcHRvciBvbiBtZW1vcnkgQiBjZWxscyBpbiBTTEU8L3RpdGxlPjxzZWNvbmRhcnktdGl0bGU+SiBFeHAgTWVkPC9zZWNvbmRhcnktdGl0bGU+PGFsdC10aXRsZT5UaGUgSm91cm5hbCBvZiBleHBlcmltZW50YWwgbWVkaWNpbmU8L2FsdC10aXRsZT48L3RpdGxlcz48cGVyaW9kaWNhbD48ZnVsbC10aXRsZT5KIEV4cCBNZWQ8L2Z1bGwtdGl0bGU+PGFiYnItMT5UaGUgSm91cm5hbCBvZiBleHBlcmltZW50YWwgbWVkaWNpbmU8L2FiYnItMT48L3BlcmlvZGljYWw+PGFsdC1wZXJpb2RpY2FsPjxmdWxsLXRpdGxlPkogRXhwIE1lZDwvZnVsbC10aXRsZT48YWJici0xPlRoZSBKb3VybmFsIG9mIGV4cGVyaW1lbnRhbCBtZWRpY2luZTwvYWJici0xPjwvYWx0LXBlcmlvZGljYWw+PHBhZ2VzPjIxNTctNjQ8L3BhZ2VzPjx2b2x1bWU+MjAzPC92b2x1bWU+PG51bWJlcj45PC9udW1iZXI+PGVkaXRpb24+MjAwNi8wOC8yMzwvZWRpdGlvbj48a2V5d29yZHM+PGtleXdvcmQ+QWR1bHQ8L2tleXdvcmQ+PGtleXdvcmQ+QWZyaWNhbiBBbWVyaWNhbnM8L2tleXdvcmQ+PGtleXdvcmQ+QW5pbWFsczwva2V5d29yZD48a2V5d29yZD5CLUx5bXBob2N5dGUgU3Vic2V0cy9jeXRvbG9neS8qaW1tdW5vbG9neS9waHlzaW9sb2d5PC9rZXl3b3JkPjxrZXl3b3JkPkNhbGNpdW0vbWV0YWJvbGlzbTwva2V5d29yZD48a2V5d29yZD5GZW1hbGU8L2tleXdvcmQ+PGtleXdvcmQ+SHVtYW5zPC9rZXl3b3JkPjxrZXl3b3JkPipJbW11bm9sb2dpYyBNZW1vcnk8L2tleXdvcmQ+PGtleXdvcmQ+THVwdXMgRXJ5dGhlbWF0b3N1cywgU3lzdGVtaWMvKmltbXVub2xvZ3k8L2tleXdvcmQ+PGtleXdvcmQ+THltcGhvY3l0ZSBBY3RpdmF0aW9uPC9rZXl3b3JkPjxrZXl3b3JkPk1hbGU8L2tleXdvcmQ+PGtleXdvcmQ+TWljZTwva2V5d29yZD48a2V5d29yZD5NaWRkbGUgQWdlZDwva2V5d29yZD48a2V5d29yZD5SZWNlcHRvcnMsIEFudGlnZW4sIEItQ2VsbC9pbW11bm9sb2d5PC9rZXl3b3JkPjxrZXl3b3JkPlJlY2VwdG9ycywgSWdHLyptZXRhYm9saXNtPC9rZXl3b3JkPjwva2V5d29yZHM+PGRhdGVzPjx5ZWFyPjIwMDY8L3llYXI+PHB1Yi1kYXRlcz48ZGF0ZT5TZXAgNDwvZGF0ZT48L3B1Yi1kYXRlcz48L2RhdGVzPjxpc2JuPjAwMjItMTAwNyAoUHJpbnQpJiN4RDswMDIyLTEwMDcgKExpbmtpbmcpPC9pc2JuPjxhY2Nlc3Npb24tbnVtPjE2OTIzODQ5PC9hY2Nlc3Npb24tbnVtPjx3b3JrLXR5cGU+UmVzZWFyY2ggU3VwcG9ydCwgTi5JLkguLCBFeHRyYW11cmFsJiN4RDtSZXNlYXJjaCBTdXBwb3J0LCBOb24tVS5TLiBHb3YmYXBvczt0PC93b3JrLXR5cGU+PHVybHM+PHJlbGF0ZWQtdXJscz48dXJsPmh0dHA6Ly93d3cubmNiaS5ubG0ubmloLmdvdi9wdWJtZWQvMTY5MjM4NDk8L3VybD48dXJsPmh0dHA6Ly9qZW0ucnVwcmVzcy5vcmcvY29udGVudC8yMDMvOS8yMTU3LmZ1bGwucGRmPC91cmw+PC9yZWxhdGVkLXVybHM+PC91cmxzPjxjdXN0b20yPjIxMTgzOTA8L2N1c3RvbTI+PGVsZWN0cm9uaWMtcmVzb3VyY2UtbnVtPjEwLjEwODQvamVtLjIwMDUxNTAzPC9lbGVjdHJvbmljLXJlc291cmNlLW51bT48bGFuZ3VhZ2U+ZW5nPC9sYW5ndWFnZT48L3JlY29yZD48L0NpdGU+PC9FbmROb3RlPn==^1^ we found that the mean fluorescence intensity (MFI) of FcγRIIb varied between B cell subpopulations in SLE. Naïve cells expressed significantly lower levels when compared with other B cell subpopulations with a hierarchy of expression: naïve < double negative < post‐switched < pre‐switched cells. Post‐switched memory cells (MCs) expressed FcγRIIb to a similar level as pre‐switched MCs and double negative cells. The horizontal line represents the median; the box, interquartile range; the whiskers, 10‐90^th^ percentile; and the dots represent outliers. (B) Naïve cells expressed significantly higher levels of IgD compared with pre‐switched cells, the results represent the mean and SD, in contrast to the expression of FcγRIIb (A).
Supplementary Figure 3. Internalization of anti‐CD20 monoclonal antibodies (mAbs) in B cell subpopulations. (A) B cell subpopulations were categorized based on the expression of CD27 and CD38. B cell subpopulations were characterized based on the expression of CD27: CD27+ or CD27‐; or the expression of CD38: CD38lo or CD38++. Surface fluorescence quenching assay was performed using enriched B cells from patients with systemic lupus erythematosus (SLE) (n=5). There was no significant difference between CD27+ and CD27‐ subpopulations in the amount of internalization of RTX or GA101_gly_. The horizontal line represents the median. (B) Similarly, there was no significant difference between in internalization of RTX or GA101_gly_ between CD38lo or CD38++ B cell subpopulations.
Supplementary Table 1. Demographics of patients with Rheumatoid Arthritis
Supplementary Table 2. Demographics of patients with Systemic Lupus Erythematosus
Supplementary Table 3. Efficiency of anti‐CD20 mAbs and frequency of B cell phenotypes of patients with Rheumatoid Arthritis and Systemic Lupus Erythematosus
