# Supplementary material for: Internalization of Rituximab and the Efficiency of B Cell Depletion in Rheumatoid Arthritis and Systemic Lupus Erythematosus
Source: Arthritis Rheumatol. 2015 Jul 28;67(8):2046–55. doi: 10.1002/art.39167 (PMC4737120; doi:10.1002/art.39167)
Supplement: Supplementary file 1 — Supplementary Figure 1. Dose‐response experiments. We determined the optimal concentration of mAbs (0.01, 0.1, 1 and 10 μg/mL) in four Independent experiments using blood from normal healthy controls and using non‐glycomodified versions of GA101 (GA101gly) to directly assess the effects of type I versus II without the influence of afucosylation. Whole blood samples were incubated with or without RTX or GA101gly at 0.01, 0.1, 1 and 10 μg/ml and percentage B cell death measured by flow cytometric analysis after 24 h and mean of triplicate wells was used. Cytotoxicity of RTX and GA101gly were compared in healthy controls (n=4). Rituximab (RTX) lyses B cells less efficiently than GA101gly in all four samples at all four concentrations tested. The results are the means and SD. Supplementary Figure 2. Differential expression of IgD and FcγRIIb in B cell subpopulations. (A) Similar to a previous report,PEVuZE5vdGU+PENpdGU+PEF1dGhvcj5NYWNrYXk8L0F1dGhvcj48WWVhcj4yMDA2PC9ZZWFyPjxSZWNOdW0+MTk1NDg8L1JlY051bT48RGlzcGxheVRleHQ+PHN0eWxlIGZhY2U9InN1cGVyc2NyaXB0Ij4xPC9zdHlsZT48L0Rpc3BsYXlUZXh0PjxyZWNvcmQ+PHJlYy1udW1iZXI+MTk1NDg8L3JlYy1udW1iZXI+PGZvcmVpZ24ta2V5cz48a2V5IGFwcD0iRU4iIGRiLWlkPSJ6eHNhejV0YWJ4ZGY1N2U1cDJocHp4NXVwMnBzZWFkNWFyc2EiPjE5NTQ4PC9rZXk+PC9mb3JlaWduLWtleXM+PHJlZi10eXBlIG5hbWU9IkpvdXJuYWwgQXJ0aWNsZSI+MTc8L3JlZi10eXBlPjxjb250cmlidXRvcnM+PGF1dGhvcnM+PGF1dGhvcj5NYWNrYXksIE0uPC9hdXRob3I+PGF1dGhvcj5TdGFuZXZza3ksIEEuPC9hdXRob3I+PGF1dGhvcj5XYW5nLCBULjwvYXV0aG9yPjxhdXRob3I+QXJhbm93LCBDLjwvYXV0aG9yPjxhdXRob3I+TGksIE0uPC9hdXRob3I+PGF1dGhvcj5Lb2VuaWcsIFMuPC9hdXRob3I+PGF1dGhvcj5SYXZldGNoLCBKLiBWLjwvYXV0aG9yPjxhdXRob3I+RGlhbW9uZCwgQi48L2F1dGhvcj48L2F1dGhvcnM+PC9jb250cmlidXRvcnM+PGF1dGgtYWRkcmVzcz5EZXBhcnRtZW50IG9mIE1lZGljaW5lLCBDb2x1bWJpYSBVbml2ZXJzaXR5IE1lZGljYWwgQ2VudGVyLCBOZXcgWW9yaywgTlkgMTAwMzIsIFVTQS4gbWNtMjEyM0Bjb2x1bWJpYS5lZHU8L2F1dGgtYWRkcmVzcz48dGl0bGVzPjx0aXRsZT5TZWxlY3RpdmUgZHlzcmVndWxhdGlvbiBvZiB0aGUgRmNnYW1tYUlJQiByZWNlcHRvciBvbiBtZW1vcnkgQiBjZWxscyBpbiBTTEU8L3RpdGx [file ART-67-2046-s001.docx]

**SUPPLEMENTARY DATA**

**Supplementary Figures**

Supplemental figure S1. Dose-response experiments. We determined the optimal concentration of mAbs (0.01, 0.1, 1 and 10 μg/mL) in four Independent experiments using blood from normal healthy controls and using non-glycomodified versions of GA101 (GA101_gly_) to directly assess the effects of type I versus II without the influence of afucosylation. Whole blood samples were incubated with or without RTX or GA101_gly_ at 0.01, 0.1, 1 and 10 μg/ml and percentage B cell death measured by flow cytometric analysis after 24 h and mean of triplicate wells was used. Cytotoxicity of RTX and GA101_gly_ were compared in healthy controls (n=4). Rituximab (RTX) lyses B cells less efficiently than GA101_gly_ in all four samples at all four concentrations tested. The results are the means and SD.

Supplemental figure S2. Differential expression of IgD and FcγRIIb in B cell subpopulations. (A) Similar to a previous report,[^1^](#_ENREF_1) we found that the mean fluorescence intensity (MFI) of FcγRIIb varied between B cell subpopulations in SLE. Naïve cells expressed significantly lower levels when compared with other B cell subpopulations with a hierarchy of expression: naïve < double negative < post-switched < pre-switched cells. Post-switched memory cells (MCs) expressed FcγRIIb to a similar level as pre-switched MCs and double negative cells. The horizontal line represents the median; the box, interquartile range; the whiskers, 10-90^th^ percentile; and the dots represent outliers. (B) Naïve cells expressed significantly higher levels of IgD compared with pre-switched cells, the results represent the mean and SD, in contrast to the expression of FcγRIIb (A).

Supplemental figure S3. Internalization of anti-CD20 monoclonal antibodies (mAbs) in B cell subpopulations. (A) B cell subpopulations were categorized based on the expression of CD27 and CD38. B cell subpopulations were characterized based on the expression of CD27: CD27+ or CD27-; or the expression of CD38: CD38lo or CD38++. Surface fluorescence quenching assay was performed using enriched B cells from patients with systemic lupus erythematosus (SLE) (n=5). There was no significant difference between CD27+ and CD27- subpopulations in the amount of internalization of RTX or GA101_gly_. The horizontal line represents the median. (B) Similarly, there was no significant difference between in internalization of RTX or GA101_gly_ between CD38lo or CD38++ B cell subpopulations.

**Reference:**

**1.** Mackay M, Stanevsky A, Wang T, et al. Selective dysregulation of the FcgammaIIB receptor on memory B cells in SLE. *The Journal of experimental medicine.* Sep 4 2006;203(9):2157-2164.

**Supplementary Tables**

Table 1. Demographics of patients with Rheumatoid Arthritis

| Patient number | RTX-CTI | GA101gly-CTI | GA101gly-CTI/ RTX-CTI | Age  (years) | disease duration  (years) | DAS-28  -ESR score | Medications |
| --- | --- | --- | --- | --- | --- | --- | --- |
| 1 | 12 | 16 | 1 | 54 | 14 | 5.2 | CS, MTX, HCQ |
| 2 | 39 | 51 | 1 | 53 | 12 | 6.2 | CS, MTX, HCQ, LFN |
| 3 | 15 | 47 | 3 | 42 | 11 | 5.99 | MTX, Humira |
| 4 | 43 | 60 | 1 | 68 | 16 | 4 | MTX |
| 5 | 15 | 21 | 1 | 62 | 9 | 4.65 | MTX |
| 6 | 20 | 74 | 4 | 35 | 2 | 5.35 | CS, MTX, HCQ, SSZ |
| 7 | 7 | 71 | 11 | 33 | 8 | 5.3 | CS, MTX, HCQ |
| 8 | 48 | 71 | 1 | 45 | 6 | 4 | SSZ, hcq |
| 9 | 17 | 65 | 4 | 49 | 14 | 3.3 | MTX, Enbrel |
| 10 | 42 | 71 | 2 | 51 | 2 | 3.5 | HCQ |
| 11 | 48 | 66 | 1 | 65 | 11 | 4.2 | MTX, HCQ, SSZ |
| 12 | 37 | 64 | 2 | 51 | 10 | 3 | CS, MTX, Ada |
| 13 | 47 | 70 | 1 | 56 | 10 | 3.5 | MTX, Toc |
| 14 | 10 | 44 | 4 | 46 | 11 | 6.3 | MTX, SSZ |
| 15 | 1 | 37 | 37 | 75 | 14 | 5.77 | CS, MTX |
| 16 | 6 | 51 | 9 | 33 | 15 | 5.9 | HCQ, Enbrel |
| 17 | 13 | 45 | 3 | 66 | 44 | 5.68 | nil |
| 18 | 53 | 84 | 2 | 56 | 3 | 5.5 | CS, MTX, SSZ, HCQ |
| 19 | 14 | 57 | 4 | 31 | 8 | 5 | MTX |
| 20 | 46 | 53 | 1 | 67 | 25 | 2.7 | nil |
| 21 | 14 | 34 | 2 | 54 | 8 | 6.38 | CS, AZA |
| 22 | 45 | 58 | 1 | 42 | 20 | 4.5 | SSZ, HCQ |
| 23 | 30 | 50 | 2 | 59 | 8 | 5.12 | CS, SSZ, HCQ |
| 24 | 29 | 46 | 2 | 79 | 1 | 5.3 | AZA |
| 25 | 30 | 60 | 2 | 50 | 14 | 4 | SSZ, MTX |
| 26 | 16 | 43 | 3 | 24 | 6 | 4.3 | MTX, SSZ |

RTX, rituximab; GA101_gly_, glycosylated GA101; CTI, cytotoxicity index; Ada, DAS-28-ESR, disease activity score-28- erythrocyte sedimentation rate; Adalimumab (anti-TNF agent); AZA, azathioprine; CS, corticosteroids; HCQ, Hydroxychloroquine; MTX, methotrexate; SSZ, sulfasalazine; Toc, Tocilizumab (anti-IL-6 receptor monoclonal antibody); Humira, anti-TNF agent; Enbrel, anti-TNF agent

Table 2. Demographics of patients with Systemic Lupus Erythematosus

| Patient  number | RTX-CTI | GA101gly-CTI | GA101gly-CTI/ RTX-CTI | age (years) | Ethn-  -icity | disease duration  (years) | Clinical  manifest-  -ations | C3  (g/L) | disease activity | BILAG score | Medications |
| --- | --- | --- | --- | --- | --- | --- | --- | --- | --- | --- | --- |
| 1 | 39 | 44 | 1.15 | 41 | AC | 7 | LN | 1.03 | inactive | 2 | AZA, HCQ |
| 2 | 18 | 46 | 2.52 | 39 | C | 23 | non-renal | 0.86 | inactive | 2 | CS, HCQ |
| 3 | 8 | 27 | 3.21 | 39 | C | 26 | LN | 1.03 | inactive | | CS, MMF |
| 4 | 10 | 47 | 4.71 | 41 | C | 23 | non-renal | 0.70 | inactive | 2 | CS, HCQ, MMF |
| 5 | 7 | 28 | 4.00 | 27 | AC | 14 | LN | 0.58 | active | 18 | CS, HCQ |
| 6 | 8 | 21 | 2.72 | 35 | C | 10 | LN | 1.00 | inactive | 0 | CS, HCQ, MMF |
| 7 | 15 | 32 | 2.20 | 34 | AC | 5 | non-renal | 0.63 | inactive | 10 | AZA |
| 8 | 29 | 52 | 1.80 | 24 | C | 11 | non-renal | 1.56 | inactive | 0 | AZA, HCQ |
| 9 | 18 | 49 | 2.72 | 42 | A | 27 | non-renal | 1.40 | inactive | 1 | CS |
| 10 | 28 | 27 | 0.96 | 53 | AC | 8 | non-renal | 0.94 | inactive | 1 | CS, HCQ |
| 11 | 15 | 49 | 3.27 | 61 | C | 12 | non-renal | 1.28 | active | 8 | CS |
| 12 | 5 | 56 | 12.31 | 39 | C | 19 | LN | 0.59 | active | 10 | CS, MMF |
| 13 | 20 | 26 | 1.31 | 22 | AC | 10 | non-renal | 0.86 | inactive | | HCQ, MMF |
| 14 | 6 | 15 | 2.50 | 52 | C | 8 | non-renal | 0.81 | inactive | 1 | CS, AZA, HCQ |
| 15 | 15 | 20 | 1.36 | 21 | AC | 4 | non-renal | 1.13 | inactive | 1 | MTX |
| 16 | 24 | 30 | 1.25 | 23 | AC | 8 | non-renal | 0.93 | inactive | 0 | HCQ, MTX |
| 17 | 22 | 46 | 2.06 | 32 | C | 2 | non-renal | 0.96 | inactive | 0 | HCQ |
| 18 | 1 | 24 | 23.84 | 39 | C | 7 | LN | 0.79 | inactive | 2 | CS, HCQ |
| 19 | 42 | 70 | 1.67 | 24 | AC | 16 | LN |  | inactive | 1 | CS, HCQ |
| 20 | 32 | 9 | 0.27 | 31 | AC | 3 | LN | 0.75 | active | 10 | CS, MMF |
| 21 | 36 | 41 | 1.14 | 76 | C | 19 | LN | 1.19 | inactive | 2 | CS |
| 22 | 30 | 58 | 1.95 | 48 | C | 16 | non-renal | 1.08 | inactive | 0 | HCQ |
| 23 | 1 | 29 | 47.20 | 39 | C | 21 | renal | 0.99 | inactive | 0 | CS, HCQ, MMF |
| 24 | 1 | 26 | 26.44 | 56 | C | 2 | non-renal | 0.80 | inactive | 1 | CS, AZA, HCQ |
| 25 | 17 | 36 | 2.12 | 41 | C | 24 | non-renal | 0.70 | inactive | 2 | CS, HCQ, MMF |
| 26 | 1 | 16 | 16.00 | 40 | AC | 7 | LN | 1.53 | inactive | 0 | CS |
| 27 | 11 | 14 | 1.31 | 29 | A | 29 | LN | 1.27 | active | 13 | CS, AZA |
| 28 | 49 | 64 | 1.33 | 24 | A | 6 | LN | 1.30 | active | 9 | CS |
| 29 | 35 | 69 | 2.01 | 26 | AC | 6 | LN | 0.91 | inactive | 2 | CS, HCQ, MMF |
| 30 | 37 | 58 | 1.57 | 51 | C | 17 | non-renal | 1.42 | inactive | 0 | NIL |
| 31 | 18 | 47 | 2.61 | 55 | C | 27 | LN | 0.60 | inactive | 0 | HCQ |
| 32 | 15 | 17 | 1.13 | 44 | C | 20 | LN | 1.10 | inactive | 0 | MTX |
| 33 | 5 | 41 | 7.55 | 46 | C | 13 | non-renal | 1.42 | active | 13 | CS, AZA, |
| 34 | 15 | 56 | 3.83 | 36 | AC | 6 | LN | 0.73 | active | 10 | CS, HCQ, MMF |
| 35 | 29 | 22 | 0.74 | 34 | AC | 18 | LN | 1.05 | inactive | 0 | CS, MMF |
| 36 | 30 | 49 | 1.65 | 32 | C | 8 | non-renal | 1.17 | inactive | 0 | HCQ |
| 37 | 16 | 66 | 4.10 | 29 | AC | 3 | non-renal | 1.14 | active | 8 | CS, HCQ |
| 38 | 12 | 36 | 3.00 | 53 | C | 4 | non-renal | 1.07 | inactive | 0 | HCQ |
| 39 | 17 | 47 | 2.76 | 23 | A | 14 | LN | 1.25 | inactive | 1 | HCQ, MMF |
| 40 | 18 | 23 | 1.26 | 49 | AC | 15 | LN | 0.80 | inactive | 2 | CS, AZA, HCQ |
| 41 | 6 | 14 | 2.51 | 39 | C | 13 | non-renal | 0.61 | inactive | 1 | CS, HCQ |
| 42 | 4 | 33 | 7.27 | 54 | A | 32 | non-renal | 1.10 | inactive | 1 | CS, HCQ |
| 43 | 13 | 38 | 2.97 | 33 | C | 18 | non-renal | 1.31 | inactive | 16 | CS, HCQ |
| 44 | 8 | 43 | 5.60 | 31 | CH | 15 | LN | 0.86 | inactive | 1 | CS, HCQ, MTX |
| 45 | 9 | 34 | 3.94 | 33 | AC | 20 | non-renal | 1.06 | active | 17 | CS, HCQ, MTX |
| 46 | 11 | 41 | 3.73 | 45 | C | 25 | non-renal | 1.08 | inactive | 1 | nil |
| 47 | 18 | 37 | 2.11 | 39 | A | 14 | non-renal | 1.0 | inactive | 0 | HCQ |
| 48 | 3 | 36 | 11.13 | 38 | C | 1 | non-renal | 1.06 | inactive | 1 | CS, HCQ |
| 49 | 26 | 38 | 1.44 | 59 | C | 20 | LN | 1.03 | inactive | 0 | CS, HCQ |
| 50 | 6 | 32 | 5.19 | 46 | C | 24 | LN | 0.94 | inactive | 1 | CS, HCQ, MMF |

RTX, rituximab; GA101_gly_, glycosylated GA101; CTI, cytotoxicity index; C3, serum complement 3; LN, lupus nephritis; non-renal, lupus manifestations not including kidney; A, Asian; AC, Afro-Caribbean; C, Caucasian; Ch, Chinese; AZA, azathioprine; CS, corticosteroids; HCQ, Hydroxychloroquine; MTX, methotrexate; MMF, mycophenolate mofetil

Table 3. Efficiency of anti-CD20 mAbs and frequency of B cell phenotypes of patients with Rheumatoid Arthritis and Systemic Lupus Erythematosus

A. Patients with Rheumatoid Arthritis

| Patient number | RTX-CTI | GA101_gly_-CTI | GA101_gly_-CTI/ RTX-CTI | Naïve  (%) | pre-switched MC (%) | post-switched MC (%) | double negative MC (%) |
| --- | --- | --- | --- | --- | --- | --- | --- |

| 1 | 15 | 47 | 3 | 64 | 4 | 26 | 7 |
| --- | --- | --- | --- | --- | --- | --- | --- |
| 2 | 7 | 71 | 11 | 71 | 9 | 11 | 9 |
| 3 | 17 | 65 | 4 | 57 | 13 | 19 | 10 |
| 4 | 10 | 44 | 4 | 58 | 21 | 7 | 15 |
| 5 | 1 | 37 | 37 | 66 | 9 | 13 | 12 |
| 6 | 13 | 45 | 3 | 69 | 4 | 13 | 15 |
| 7 | 53 | 84 | 2 | 67 | 6 | 16 | 11 |
| 8 | 14 | 57 | 4 | 59 | 8 | 11 | 23 |
| 9 | 12 | 26 | 2 | 68 | 7 | 12 | 13 |

| 10 | 16 | 43 | 3 | 61 | 6 | 24 | 9 |
| --- | --- | --- | --- | --- | --- | --- | --- |
| B. Patients with Systemic Lupus Erythematosus | | | | | | | |

| 1 | 10 | 47 | 5 | 51 | 3 | 19 | 28 |
| --- | --- | --- | --- | --- | --- | --- | --- |
| 2 | 29 | 52 | 2 | 59 | 19 | 8 | 15 |
| 3 | 15 | 49 | 3 | 70 | 10 | 11 | 9 |
| 4 | 6 | 15 | 3 | 72 | 5 | 15 | 8 |
| 5 | 15 | 17 | 1 | 73 | 3 | 13 | 11 |
| 6 | 5 | 41 | 8 | 66 | 7 | 15 | 13 |
| 7 | 13 | 38 | 3 | 73 | 2 | 14 | 11 |
| 8 | 3 | 36 | 11 | 69 | 3 | 16 | 12 |
| 9 | 6 | 32 | 5 | 73 | 8 | 9 | 10 |

mAb, monoclonal antibody; RTX, rituximab; GA101_gly_, glycosylated GA101; CTI, cytotoxicity index; naïve, IgD+CD27-;

Pre-switched MC (memory cells), IgD+CD27+; post-switched MC, IgD-CD27+; double negative MC; IgD-CD27-
